# CGRP Receptor Antagonists and 5-HT1F Receptor Agonist in the Treatment of Migraine

**DOI:** 10.3390/jcm10071429

**Published:** 2021-04-01

**Authors:** Matilde Capi, Valerio De Angelis, Donatella De Bernardini, Ottavia De Luca, Fabiola Cipolla, Luana Lionetto, Maurizio Simmaco, Paolo Martelletti

**Affiliations:** 1 Laboratory of Clinical Biochemistry, Mass Spectrometry Unit, Sant’Andrea University Hospital, 00185 Rome, Italy; matildecapi@gmail.com (M.C.); donatelladb@yahoo.it (D.D.B.); luanalionetto@gmail.com (L.L.); maurizio.simmaco@uniroma1.it (M.S.); 2 Department of Clinical and Molecular Medicine, Sapienza University of Rome, 00185 Rome, Italy; valerio.deangelis@uniroma1.it (V.D.A.); fabiola.cipolla@alice.it (F.C.); 3 Laboratory of Clinical Biochemistry, Advanced Molecular Diagnostic Unit, Sant’Andrea University Hospital, 00185 Rome, Italy; ottavia_deluca@yahoo.it; 4 Department of Neurosciences, Mental Health and Sensory Organs (NESMOS), Sapienza University, 00185 Rome, Italy

**Keywords:** migraine, CGRP, therapy, gepants, ditans, antagonism, CGRP receptor

## Abstract

Discovering that calcitonin-related peptide (CGRP) plays a key role in the complex pathophysiology of migraine has allowed us to make great strides in the development of new approaches for acute and preventive treatment. This evidence has led to the development of small molecules antagonist molecules of the CGRP receptor (“gepants”) and of a new class of medications called “Ditans”. This review presents the data from clinical trials reporting the efficacy, safety, and tolerability of the new drugs used in the treatment of migraines. Evidences show that therapeutic approaches targeted to CGRP have the potential to transform the clinical management of migraine, even though its appropriate place has yet to be determined with accuracy.

## 1. Introduction

Migraine is not a symptom, but a complex neurological disease which, according to the World Health Organization, represents the third most frequent disease under the age of 50. A study carried out in 2016 by the Global Burden of Disease (GBD) classifies migraine as the sixth most widespread and one of the main causes of disability worldwide, as it often manifests in the working ages, in young adults and in women of childbearing age [1]. It represents, therefore, a real social problem, in terms of years of life lived with a disability, which especially afflicts individuals aged 15 to 49. Migraine symptoms include pain, sensitivity to light, sound, odors, and changes in vision, in addition to nausea, vomiting, tingling, and speech disorders, and have significant disabling effects on the physical, social, and professional functioning of the patient [2]. Since the first drug for the treatment of migraine was introduced, other medications including calcium channel antagonists, antidepressants, antiepileptics, and antihypertensives developed for indications other than migraine, have entered the field based on clinical studies. However, efficacy is often insufficient, and the incidence of adverse effects leads to the suspension of the therapeutic treatment [3]. The discovery of the key role of the peptide related to the gene of calcitonin (CGRP) in the complex pathophysiology of the migraine has made it possible to make large progress in developing new approaches to preventive and acute treatment [4]. This has led to the development of small molecules antagonist of the CGRP receptor (“gepants”) and monoclonal antibodies (mAbs) affecting the CGRP or its receptor. In studies on monoclonal antibody therapies, no serious safety or tolerability problems have been identified. These monoclonal antibodies are the first specific treatment for the prevention of migraine, and they have good efficacy and tolerability [5]. In addition, thanks to the long half-life, their allow treatment schemes that are well accepted by the patient, with one administration every 4 or 12 weeks [6]. All monoclonal antibodies acting on the CGRP have been shown to modify and improve quality of life (Qol) and headache-related disability. Erenumab, Galcanezumab, and Fremanezumab have been approved both by the US Food and Drug Administration (FDA) and the European Medicine Agency (EMA) for migraine treatment; the latest (Eptinezumab) was approved by the FDA in February 2020. Another valid support in the pharmacological treatment of migraine has been given by the development of the gepants.

Gepants, selective antagonists of the CGRP receptor, are small molecules that compete with endogenous CGRP at a specific receptors level. Some years ago, first generation gepants like olcegepant, telcagepant, BI44370TA, and MK-3207 proved to have good efficacy but their development was discouraged because of a scarcely favorable safety profile and the route of administration, only intravenous [7]. The search for new gepants thus focused on molecules that did not present chemical structures potentially related to hepatotoxicity problems. Studies led to the development of two new candidates, rimegepant and ubrogepant, recently approved by the FDA for the treatment of acute migraine headache, while atogepant remains an experimental drug for preventive therapy. Vazegepant, with positive preliminary data, would be the first intranasal gepant for acute treatment. Further studies are needed in order to define a safety and efficacy and tolerability profile for new pharmacological treatments but the new therapies available up to date consent to approach the precision medicine for migraine, allowing specific therapies for individual patients. Recently new molecules called “Ditans” have been developed; they are selective agonist for 5-HT1F receptor with a better tolerability profile compared to triptans, due to their very low affinity for 5-HT1B-1D receptors subtypes, known for their involvement in vasoconstrictive mechanisms such as Lasmiditan [8].

## 2. Methods

This literature review research was carried out in November 2020 on MEDLINE (www.ncbi.nlm.nih.gov/pubmed, last accessed on 20 March 2021) for both original and review papers on the mechanism of action of CGRP antagonists and 5-HT1F agonists in migraine, and for randomized double-blind placebo-controlled trials on the new generation of Gepants and Ditans until 2020. For the purpose of this review, we also performed research on www.clinicaltrials.gov and on The International Register of Clinical Trials Portal (www.who.int/ictrp, last accessed on 30 November 2020) for named Gepants and Ditans. We considered only outcomes from randomized controlled trials (RCTs) of adult subjects affected with migraine with or without aura and studies describing the pharmacological treatment of migraine using CGRP Receptor Antagonists and the 5-HT1F receptor agonist. We excluded studies not regarding migraine or involving people under 18 years, studies having an unclearly defined design, target population, and/or results, all the open label studies or trials that did not have placebo controls, and all the studies in healthy subjects. We limited our search to articles regarding human subjects and published in English.

## 3. Molecular Mechanisms of CGRP Antagonists in Migraine

Many studies have hypothesized the key role played by the CGRP in the pathophysiology of migraine, confirmed over the years by clinical findings even if its specific pathogenic mechanisms in migraine is still being investigated [9]. In these studies, CGRP is found to be released from trigeminal ganglia neurons, its transcription increases under conditions imitating neurogenic inflammation, and migraine pharmacotherapies can both inhibit CGRP transcription and reduce CGRP release, and tumor necrosis factor-α (TNF-α), an endogenous inflammatory mediator involved in migraine, can arouse CGRP transcription [9]. CGRP is a neuropeptide consisting of 37 amino acids and belongs to a family of peptides that includes calcitonin (CT), adrenomedullin (AM), and amyl (AMY). This peptide presents itself in α and β isoforms, produced by two different genes (respectively CALC I and CALC II) located in two different sites of the same chromosome: chromosome 11 [10]. These two isoforms, in human, differ by three amino acids and perform similar biological activities. The α isoform is considered the main neuronal form, being located at the level of sensory nerve endings of the central and peripheral nervous system; β-CGRP was initially found in the enteric nervous system, while recent evidence shows that β isoform may be released together with α-CGRP at the vascular level [11]. The CGRP receptor, related to a protein G, is formed by three subunits: receptor activity-modifying protein 1 (RAMP1), calcitonin-like receptor (CLR), and the component protein receptor (CPR) [12]. The mechanism by which CGRP binds to the receptor can be interpreted according to the general model of the two domains indicated by Hoare. In this model, the C-terminal of the peptide ligand firstly binds with high affinity to the extracellular portion N-terminal receptor forming a so-called affinity trap [13]. This initial event increases the local peptide concentration, which then allows for the N-terminal portion of the CGRP to interact with the juxtamembrane region of the CLR, thus activating the receptor and determining the accumulation of cyclic adenosine monophosphate (Camp) [14]. The activation of CGRP receptors regulates the increase of intracellular levels of cAMP and cyclic guanosine monophosphate (cGMP) in numerous body cells and these are the second messengers associated with vasodilation. The role of CGRP in vasodilation of peripheral vascular beds can be described through many vascular mechanisms. To date, the CGRP, when it binds to its receptor, induces a vasodilation through an endothelium-dependent and endothelium-independent mechanism [15]. In ats and humans’ arteries level the CGRP, in its independent endothelium path, binds directly to the CGRP receptors on smooth muscle cells activating adenylate cyclase. This process produces an increase in cAMP levels resulting in vascular relaxation. CGRP also has the ability to stimulate the production of NO by acting via a receptor located on the endothelium (pathway dependent endothelium). In this way, the CGRP binds to its receptor and causes an increase in cAMP through adenylate cyclase. This process determines the activation of the enzyme NO synthetase (NOS) which leads to an increase in nitrogen oxide levels (NO). Then, NO activates the route of guanylate cyclase on smooth muscle cells leading to the production of cGMP and vascular relaxation [16]. The first person to recognize the implication of GCRP in migraine was Edvinsson in 1990 when he demonstrated its role in the cerebrovascular trigeminal neuron system as vasoactive constituent and therefore implied in the cerebral vasoconstriction processes [17]. The recognition of the important role of the CGRP in the pathogenesis of migraine has aroused great interest in the study of new therapeutic approaches. The CGRP receptor antagonists have shown a good clinical efficacy in the treatment of acute migraine attacks and offer a valid alternative to current therapies.

## 4. Preventative New Gepants

Preventive treatment therapy aims to lower the frequency, intensity, and duration of migraine headaches increasing the efficacy of drugs and reducing the risk of worsening and the chronicization of the headache. It also aims to reduce symptomatic drugs assumption. Preventive treatment is efficacious when at least one of the following conditions occurs: a 50% reduction in the frequency of migraine days, a significant reduction in migraine attacks or their severity or an increased response to therapeutic treatment, a reduction in disability due to the disease, or an improvement in Qol [18]. Preventative treatment drugs vary from antiepileptics to calcium channel blockers or angiotensin converting enzyme inhibitors. It is very important to choose the right treatment for each individual, depending on the efficacy and side-effect profile and the patient’s comorbidities or preferences [19]. Seven gepants have been developed since 2004. Gepants are small molecule calcitonin gene-related peptide (CGRP) receptor antagonists and will be prescribed for a number of patients who found other therapies ineffective or with present cardiac or cerebrovascular risks factors.

### 4.1. Atogepant

#### 4.1.1. Efficacy

Atogepant, a gepant discovered by Merck Sharp & Dohme, was the first oral CGRP antagonist developed for the preventative treatment of migraine. To date, A Phase 2/3, Multicenter, Randomized, Double-Blind, Placebo Controlled, Parallel-Group Study was conducted to evaluate efficacy, safety, and tolerability of multiple dosing regimens of oral AGN-241689 in episodic migraine prevention (NCT02848326) [20]. 834 participants with migraines, with or without aura, were given once daily (QD) atogepant 10 mg (*n* = 93), 30 mg (*n* = 183) 60 mg (*n* = 186) and twice daily (BID) 30 mg (*n* = 86) and 60 mg (*n* = 91) respect to placebo group (*n* = 186). The mean of monthly migraine days (MMD) at baseline was 7.67 ± 2.49. Regarding the efficacy, results showed a reduction from baseline in MMD on average compared to the placebo group. Particularly, the MMD change was −2.85 (placebo group), −4.0 (10 mg QD, *p* = 0.024), −3.76 (30 mg QD, *p* = 0.039), −3.55 (60 mg QD, *p* = 0.039), −4.23 (30 mg BID, *p* = 0.003) and −4.14 (60 mg BID, *p* = 0.003). A further analysis of the data showed that the least squares mean was −2.93 for placebo and −4.31 (10 mg QD), −4.17 (30 mg QD), −4.23 (30 mg BID), −3.86 (60 mg QD) and −4.32 (60 mg BID). A secondary endpoint was the proportion of patients who achieved at least a 50% reduction in mean monthly migraine days. The data reported a reduction of 40.4% (placebo group), 57.6% (atogepant 10 mg QD), 53.3% (atogepant 30 mg QD), 58.2% (atogepant 60 mg QD), 52.0% (atogepant 30 mg BID) and 62.1% (atogepant 60 mg QD). The change of least squares mean was −2.42 (placebo group), −3.71(10 mg QD), −3.86 (30 mg QD), −3.53(60 mg QD) it −3.77 (30 mg BID) it was −3.64 (60 mg BID).

#### 4.1.2. Safety and Tolerability

In a phase 2/3, multicenter, randomized, double-blind, placebo controlled, parallel-group study (NCT02848326) assessed the safety profile of atogepant. The most adverse events (AEs) were nausea, constipation, nasopharyngitis, and urinary tract infection (frequency ≥ 5%) in all groups compared to placebo. Alanine transaminase or aspartate aminotransferase were found >3× the upper limit normal (ULN) in 1.7% (placebo group), 2.2% (10 mg QD), 0.6% (30 mg QD), 1.7% (60 QD), 1.2% (30 mg BID) and 1.1% (60 mg BID). The results indicate that atogepant is well tolerated and drug-related serious AEs were absent [21].

## 5. Acute New Gepants

The acute treatment of migraine attacks provides numerous options for choosing the drug to use, but unfortunately still none of them is proven to be effective, in equal measure, for all migraine patients. Therapy must be personalized and cannot predict which type of acute therapy will be effective for a specific patient and therefore it is of-ten necessary to try with various types of molecules before finding the most suitable one. A drug therapy effectiveness determines an improvement of Qol, greater adherence to therapy and treatment in general for its part as well as reducing the risk of chronicization and abuse of the symptomatic drugs [22]. The two main categories of drugs with evidence of efficacy in the acute treatment of migraine attack remain today the triptans and the nonsteroidal anti-inflammatory drugs (NSAIDs). Up to date, FDA has approved many drugs for migraine acute treatment in adults, but their efficacy is not homogeneous and their use can also be limited by safety concerns [23].

### 5.1. Ubrogepant

#### 5.1.1. Efficacy

Ubrogepant (MK-1602) is a novel oral CGRP receptor antagonist, and it has been developed for the acute treatment of migraine. Its absorption in humans is rapid and its apparent half-life is about three h. Its metabolization pathway goes through the liver, principally via CYP3A4, and it is also a *p*-glycoprotein substrate [24]. Two randomized, double-blind, placebo-controlled trials [<ACHIEVE I (NCT02828020) and ACHIEVE II (NCT02867709)] were carried out in order to assess the efficacy of UBRELVY for the acute treatment of migraine.

In ACHIEVE I, ubrogepant 50 mg, 100 mg or placebo were administered to 1327 patients. Pain freedom at 2 h was 19.2% (vs. placebo, *p* = 0.0023) in the 50 mg group, 21.2% (vs. placebo, *p* = 0.0003) in the 100 mg group and 11.8% in the placebo group, while freedom from most bothersome symptom (MBS) at 2-h was reported by 38.6% in the 50 mg group (vs. placebo, *p* = 0.0023) and 37.7% in the 100 mg group (vs. placebo, *p* = 0.0023) compared to 27.8% for placebo, respectively [25].

The ACHIEVE II trial was carried out on 1686 patients administered respectively with ubrogepant 25 mg, ubrogepant 50 mg or placebo. Pain freedom at 2 h was 20.7% (vs. placebo, *p* = 0.0285) in the 25 mg group, 21.8% (vs. placebo, *p* = 0.0129) in the 50 mg group and 14.3% in the placebo group, while freedom from MBS at 2-h was reported by 34.1% of the 25 mg group (vs. placebo, *p* = 0.0711) and 38.9% of the 50 mg group (vs. placebo, *p* = 0.0129), compared to 27.4% for placebo. The 25 mg dose was not statistically significant compared to placebo [26].

Another multicenter, randomized, open-label extension study, UBR-MD-04, included 1254 patients who completed one between ACHIEVE I (NCT02828020) or ACHIEVE II (NCT02867709) trials. These patients were administered with both 50 mg and 100 mg doses and they could take a second dose or a rescue medication if they did not respond to the first dose or if they experienced a migraine recurrence. The effects of ubrogepant were observed in the short long-term ACHIEVE clinical trial (NCT02873221). These studies were aimed to assess the long-term safety and tolerability of discontinuous treatment with ubrogepant for the acute treatment of migraine (with or without aura) over 1 year. 1254 subjects were enrolled to evaluate the efficacy of 50 mg (*n* = 417) and 100 mg (*n* = 420) dose of ubrogepant respect to a standard therapy prescribed by the physician (*n* = 417) [27]. Results after one year reported pain freedom at 2 h after the initial dose in ≈24% of ubrogepant-treated (50 or 100 mg) attacks and pain relief at 2 h post-dose in ≈67% of ubrogepant-treated attacks [28]. 

A phase IIb, multicenter, randomized, double-blind, placebo-controlled trial (NCT01613248) was conducted to investigate the efficacy and safety of ubrogepant [29]. In this study, 834 subjects with migraine disease were enrolled and randomized to ubrogepant 1 mg (*n* = 138), 10 mg (*n* = 139), 25 mg (*n* = 139), 50 mg (*n* = 139), 100 mg (*n* = 140) respect to placebo group (*n* = 139). The percentual of Reporting Pain Freedom (PF) at 2 h post-dose was 5.6 (1 mg dose), 14.8% (10 mg), 21.4% (25 mg), 21% (50 mg), 25.5% (100 mg) respect to 8.9% (placebo). The percentage of participants reporting pain relief (PR) at 2 h post-dose was 37.4% (1 mg dose), 52.8% (10 mg), 53.4% (25 mg), 57.1% (50 mg), 58.8% (100 mg) respect to 44.6% (placebo). The percentage of Participant Reporting Absence of Photophobia at 2 h post-dose was 37.4% (1 mg dose), 43.5% (10 mg), 39.8% (25 mg), 47.6% (50 mg), 54.9% (100 mg) respect to 30.4% (placebo). Significant differences respect to placebo group were also seen for ubrogepant 50 mg regarding to absence of phonophobia and photophobia at 2 h, sustained pain freedom and sustained pain relief 2–24 and 2–48 h, total migraine freedom at 2 h and at a 2–24 h. Significant data were found for ubrogepant 25 mg regarding a sustained pain freedom 2–24 h and 2–48 h, for sustained pain relief 2–48 h and for total migraine freedom at 2 h [30].

#### 5.1.2. Safety and Tolerability

In the ACHIEVE I and II studies, the AEs most commonly reported were nausea, somnolence, and dry mouth with a frequency lower than 5% (ACHIEVE I) and lower than 2.5%, (ACHIEVE II). Hepatotoxicity was not reported [25,26].

In a randomized, open-label extension study (NCT02873221), the treatment related AEs were reported by the 10.4% and 10.5% of the ubrogepant 50 mg and 100 mg groups, respectively. One patient in the ubrogepant 50 mg group, with a previous history of supraventricular tachycardia with ablation, experienced a serious AEs (sinus tachycardia) that the investigator considered treatment related. The results also reported 20 cases of AST/ALT levels ≥3 × ULN in the two groups, and two of these cases in the 50 mg group were considered treatment related, while one in the 100 mg group was considered only probably related to the presence of confounding factors [28].

In a phase IIb, multicenter, randomized, double-blind, placebo-controlled trial (NCT01613248) the reporting absence of nausea at 2 h post-dose was 59.8% (1 mg dose), 67.6% (10 mg), 73.8% (25 mg), 68.6% (50 mg), 70.6% (100 mg) respect to 62.5% (placebo). The incidences of AEs in the first 48 h after administration were similar for the ubrogepant groups and were mostly dry mouth, nausea, fatigue, dizziness, and somnolence that did not appear to be dose dependent. There were no changes in liver enzymes or laboratory values. An increase of AST > 3 occurred in the 50 mg group but this event was declared unrelated to the treatment [30].

### 5.2. Zavegepant

#### 5.2.1. Efficacy

Biohaven Pharmaceuticals developed Zavegepant (synonyms BHV-3500), a calcitonin gene-related peptide (CGRP) receptor antagonist, for the treatment of migraine. It is administered intranasally and is currently undergoing clinical trials. A phase 2 and 3 multicenter, randomized, double-blind, placebo controlled clinical trial (NCT03872453) was recently carried out on safety and efficacy of three different intranasal dose levels of BHV-3500 for the acute treatment of migraine. Patients enrolled (*n* = 2154) have had from 2 to 8 moderate to severe migraines per month during the previous 3 months, suffering from migraine for more than 1 year with an onset prior to 50 years of age. They received BHV-3500 5 mg, 10 mg and 20 mg matching placebo. Pain relief at 2 h was respectively 19.6% (*p*-value = 0.1214), 22.5% (*p*-value = 0.0113) and 23.1% (*p*-value = 0.0055) compared to placebo group (*n* = 401, 15.5%) for the groups treated with vazegepant 5 mg (*n* = 387), 10 mg (*n* = 391) and 20 mg (*n* = 402) [31].

Treatment with BHV-3500 showed a freedom from MBS in 39.0% subjects treated with 5 mg (*p*-value: 0.1162), 41.9% in subjects treated with 10 mg (*p*-value: 0.0155) and 42.5% in subjects treated with 20 mg (*p*-value: 0.0094), respectively, compared to placebo (33.7%) [32].

#### 5.2.2. Safety and Tolerability

To date, there are no available data on tolerability. The pre-clinical data of Intranasal vazegepant reported by Biohaven showed a good tolerability in the single dose trial. The AEs in more than 5% of the treated subjects were dysgeusia (13.5 to 16.1% vazegepant, 3.5% placebo) and nasal discomfort (1.3 to 5.2% vazegepant, 0.2% placebo), the majority of them (>80%) being of mild intensity. Data on the patients treated with 5 mg (*n* = 388), 10 mg (*n* = 394), 20 mg (*n* = 403) and placebo group (*n* = 403) reported no signal of hepatoxicity as no AST or ALT > 3× ULN, or total bilirubin > 2× ULN [32].

### 5.3. Rimegepant

#### 5.3.1. Efficacy

Rimegepant is a calcitonin-related peptide receptor antagonist which was found to be effective in the treatment of acute migraine in its oral administration, especially in those patients whose symptoms do not respond to triptans. Unlike triptans, rimegepant has no vasoconstrictor effects and is not contraindicated in patients with cardiovascular disease. Efficacy, safety and tolerability have been demonstrated by several clinical studies. A Phase 2 and 3 studies (NCT03235479 and NCT03266588) recruited 1485 subjects and 3019 subjects respectively [33,34]. In the phase 3 study, 75 mg of rimegepant dose were administered to patients in respect to a placebo group. The primary outcomes have been analyzed were freedom from pain at 2 h post-dose, freedom from MBS at 2 h post-dose while the secondary outcomes included a several parameter such as photo-phobia-free, pain relief, nausea-free, rescue medications sustained pain free and pain re-lapse at 2 h or 24–48 h. The results showed a significant and durable clinical effect with a single dose of rimegepant in pain freedom, freedom from MBS, pain relief and recovery of normal function and other outcome measures.

A phase IIb, double-blind, randomized, placebo controlled, dose-ranging trial (NCT01430442) evaluate the efficacy of rimegepant (BMS-927711) compared with placebo in the acute treatment of migraine. A group of 1026 participants received one dose of rimegepant (among the 10 mg, 25 mg, 75 mg, 150 mg, 300 mg, or 600 mg) or placebo or sumatriptan (100 mg). More patients in the rimegepant 75 mg (31.4%, *p* = 0.002), 150 mg (32.9%, *p* < 0.001), and 300 mg (29.7%, *p* = 0.002) groups and the sumatriptan group (35%, *p* < 0.001) reached freedom from pain at two h post dose vs. placebo (15.3%), and this result was significant. The secondary endpoint, sustained pain freedom from two to 24 h post-dose, was significantly reached from rimegepant doses (25–600 mg) compared to placebo [35].

A phase 3 (NCT03237845) evaluates the efficacy of rimegepant compared with placebo in migraine acute treatment. 1499 participants were enrolled to receive to rimegepant 75 mg oral tablet, or placebo 75 mg oral tablet. All the primary endpoints were met, and most patients achieved pain relief, the benefit was durable (24 and 48 h), achieved normal function and used a lower number of rescue meds [36].

A double-blind, randomized, placebo-controlled, multicenter phase 3 clinical trial (NCT03461757) evaluated the efficacy of rimegepant in subjects with Acute Migraines respect to placebo group. 1811 participants were enrolled to receive 75 mg of Rimegepant dose respect to placebo. The results showed that rimegepant orally disintegrating tablet was superior to placebo at 2 h post-dose in freedom from pain (21% vs. 11%, *p* < 0.0001) and freedom from the MBS (35% vs. 27%, *p* = 0.0009) [37].

#### 5.3.2. Safety and Tolerability

Regarding AEs, phase 2 and 3 clinical trials (NCT03235479 and NCT03266588) showed that rimegepant 75 mg oral tablet proved to be tolerable, safe and comparable to placebo on tests of liver function. The majority of AEs reported in NCT03266588 were of mild or moderate in intensity, being the most common upper respiratory tract infection (8.5%), nasopharyngitis (6.4%), and sinusitis (4.8%) [33,34].

In a Phase IIb, double-blind, randomized, placebo controlled, dose-ranging trial (NCT01430442), the most common AE observed in the rimegepant dosing groups was nausea, which was found to be dose dependent: 1.4% in the 10 mg; 0 in the 25 mg; 3% in each of the 75 mg and 150 mg dose groups; 4% in the 300 mg; and 8% in the 600 mg group [35].

A randomized, phase 3 clinical trial (NCT03237845) reported that the common AEs in 75 mg rimegepant dose were nausea (1.8%), urinary tract infection (1.5%). Safety and tolerability profiles were similar to placebo and better if compared to the past triptan experience [36].

In a randomized controlled trial (NCT03461757) no serious AEs were reported. The most common were nausea (2% in rimegepant vs. 1% in placebo group) and urinary tract infection (1% in rimegepant dose vs. 1% in placebo group). Two participants, one for each group, reported abnormal transaminase concentration of more than 3× the upper limit and it was not related to study medication. Moreover, no elevations in bilirubin greater than 2 times the upper limit of normal were reported [37].

## 6. 5-HT1D Agonists–Ditans

Numerous studies on the pathophysiology of migraine have shown the crucial im-portance of the serotoninergic system in the genesis of the attack that, and this has led to the development of 5-HT1 receptor agonists, including 5-HT1B/D and 5-HT1F, in order to achieve a better treatment for this burdensome disease.

### 6.1. Molecular Mechanisms of 5-HT1F Receptor Agonist

When the serotoninergic receptor 5HT1B-1D is activated it induces vasoconstriction, neurogenic inflammation blockage, and a reduction of cephalalgic symptoms. These effects produce a risk for patients with cardiovascular and/or cerebrovascular diseases [38]. Recently, new molecules have been developed called “Ditans”. These molecules are selective agonist for 5-HT1F receptor and have a low affinity with the 5-HT1B-1D receptors subtypes, giving them a better tolerability profile compared to triptans with a lower involvement in vasoconstrictive mechanisms [39]. It is assumed that the activation of the 5-HT1F receptor inhibits trigeminal impulses hyperpolarizing nerve terminals, but not many studies have investigated its downstream pathways. Among the 5-HT1F receptor agonists being developed, only one molecule entered a clinical trial so far: LY334370, a molecule with a 100-fold higher selectivity for the 5-HT1F receptor than the 5-HT1B and 5-HT1D receptors [40].

### 6.2. Lasmiditan

#### 6.2.1. Efficacy

For acute migraine therapy with or without aura, the FDA approved the drug Lasmiditan (previously known as COL-144 and LY573144) under the trade name Reyvow (Eli Lilly, Indianapolis, Indiana, USA) and is available in tablets of 50 mg and 100 mg. This drug has a different chemical structure from other compounds and is part of a new class of drugs called “Ditans”. Lasmiditan is a new receptor agonist 5-HT1F and the activation of 5-HT1F receptors decreases the expression of c-Fos, a neural activation marker, in the caudate nucleus of the trigeminum, without having any vascular effects [41]. Two randomized, double-blind, placebo-controlled trials [SAMURAI (NCT02439320) and SPARTAN (NCT02605174)] were con-ducted to evaluate the efficacy of lasmiditan in the acute treatment of migraine, enrolling patients with a migraine history according to the criteria of the International Classification of Headache Disorders (ICHD-II) [42,43]. The primary objective of each trial was to evaluate the efficacy of lasmiditan vs placebo in treating migraine-related headache pain and MBS. SAMURAI randomized patients to lasmiditan 100 mg (*n* = 744), or 200 mg (*n* = 745) or placebo (*n* = 742) [26]. The patients recruited for the two trials were enlisted in the open GLADIATOR (NCT02565186) study, conducted up to 12 months. These clinical trial randomized patients to lasmiditan 100 mg (*n* = 1046), or 200 mg (*n* = 1125) or placebo (*n* = 2171) in order to assess safety, efficacy and tolerability of long-term intermittent use of lasmiditan 100 mg and 200 mg for the acute treatment of migraine [44]. Its long-term intermittent use for the acute treatment of migraine [44]. In the SAMURAI clinical trial, pain freedom at 2 h was observed in 28.2% (vs. placebo, *p* < 0.001) in the 100 mg group, 32.2% (vs. placebo, *p* < 0.001) in the 200 mg group and 15.3% in the placebo group. Freedom from most bothersome symptom at 2 h was 40.9% (vs. placebo, *p* < 0.001) in the 100 mg group, 40.7% (vs. placebo, *p* < 0.001) in the 200 mg group compared to 29.5% in the placebo group. The most common adverse events were dizziness and paresthesia, both from mild to moderate intensity. The SPARTAN clinical trial showed similar data, proving a good efficacy also for the 50 mg group: pain freedom at 2 h was observed in 28.6% (vs. placebo, *p* = 0.003) in the 50 mg group, 31.4% (vs. placebo, *p* < 0.001) in the 100 mg group, 38.8% (vs. placebo, *p* < 0.001) in the 200 mg group and 21.3% in placebo group. Freedom from most bothersome symptoms at 2 h occurred in 40.8% (vs. placebo, *p* = 0.009) in the 50 mg group, 44.2% (vs. placebo, *p* < 0.001) in the 100 mg group, 48.7% (vs. placebo, *p* < 0.001) in the 200 mg group and 33.5% in the placebo group. Adverse events reported were dizziness, paresthesia, somnolence, fatigue, nausea and lethargy.

A recent phase 2 clinical trial (NCT00883051) evaluate the safety and efficacy of treating migraine headache of a series of oral doses of COL-144, in order to select a dose or doses for further evaluation. These clinical trial randomized patients to lasmiditan 50 mg (106), 100 mg (*n* = 104), 200 mg (*n* = 100), 400 mg (*n* = 99) and placebo (*n* = 103). Lasmiditan 50, 100, 200, or 400 mg doses were significantly superior to placebo in reducing migraine pain intensity, and this was especially true for lasmiditan 200 mg (19%, *p* = 0.032) and 400 mg (28%, *p* = 0.0007) [45,46]. All the efficacy results reported in this review are summarized in Table 1.

#### 6.2.2. Safety and Tolerability

In both these studies (NCT02439320 and NCT02605174) lasmiditan proved its safety and no serious AEs were reported [26].

In an open-label, long-term, safety study of Lasmiditan (NCT02565186), the most frequent treatment-emergent adverse events (TEAEs) (>2%) reported were dizziness (18.6%), somnolence (sleepiness or drowsiness; 8.5%), paresthesia (tingling or numb sensation on the skin; 6.8%), fatigue (5.5%), nausea (4.7%) and asthenia (physical weakness or lack of energy; 2.0%) [47].

In a double blind, randomized, placebo-controlled, parallel group dose-ranging study of oral COL-144 in the Acute Treatment of Migraine (NCT00883051), the AEs reported were dizziness, fatigue, vertigo, paresthesia, and somnolence [48]. All the clinical studies analyzed in this review are summarized in Table 2.

## 7. Discussion

The pharmacological treatments currently available for migraine headaches are able to provide complete pain relief and, sustained within 2 h, only in a small part of patients. Very often, the excessive use of these medicines is associated with the onset of headaches from abuse or overuse medication overuse. To date, the ineffectiveness of the pharmacological treatments is also impeded by the discontinuity due to the poor tolerability of some drugs, or the contraindications for the presence of cardiovascular disorders or significant risks. In recent years, clinical and preclinical research has largely treated the pathophysiological basis of migraine, and calcitonin-gene related peptide (CGRP) seems to play a crucial role in migraine attacks. The CGRP was initially de-scribed as a powerful mediator able to perform different functions in the nervous system, intestine and heart. Subsequently, his role as a vasodilator was discovered at the level of the cerebral and peripheral circulation. Therefore, CGRP is a valid therapeutic target and “Gepants” development represents a great opportunity for the anti-migraine therapies field. Gepants, selective CGRP receptor antagonists, are small molecules that compete with endogenous CGRP at the level of specific receptors. The gepants of first generation, olcegepant, telcagepant, BI44370TA and MK-3207, had shown a few years ago a good effectiveness but their development had been discouraged by an unfavorable safety profile or the need for its intravenous route of administration. In recent years, further clinical studies have been carried out on new molecules at different therapeutic doses and all the gepants appear to have similar effectiveness, minimal side effects, and to be safe. CGRP receptor antagonists are the first non-serotoninergic drugs specific for migraine without a vasoconstrictive action. Therefore, they could be indicated in patients with vascular diseases as peripheral vascular disease and coronary artery disease. Another pharmacological class in clinical trials is the ditans, serotoninergic agonists with specific affinity towards 5-HT1F receptors, are another pharmacological class investigated in clinical trials. Among the ditans, the molecule currently under study for the acute treatment of migraine is lasmiditan, that showed good efficacy and tolerability in two randomized and controlled studies (intravenous and oral administration) and was approved by the FDA in October 2019.

## 8. Conclusions

The new pharmacological treatments available today for the acute or preventive treatment of migraine are a huge step forward in the management of the disease. In particular, the options for a specific treatment for migraine are now strongly increased since the introduction of many new approaches to the peptide related to the CGRP receptors. In this sense, especially the introduction of specific monoclonal antibodies for migraine has represented a big step forward as well as the development of the new molecules acting as antagonists for the CGRP receptors. In the near future, the introduction on large scale of the CGRP agonists will provide a better tool for migraine therapies. The introduction of specific preventive therapies for migraine headaches based on sound science is an encouraging development for patients, in addition to the possibility of a significantly improved quality of life for those who respond to new treatments. These new therapies will allow to get a step closer to precision medicine for migraine, allowing a specific therapy for any individual patient. This could definitely improve the care of all migraine patients worldwide.

## Figures and Tables

**Table 1:** Gepants and ditans efficacy data.

Drug	Identifier	Intervention	Monthly Migraine Days	50% Reduction in Mean MMD	Least-Squares Means	Pain Free at 2 h	Bothersome Symptoms (MBS)	Pain Relief at 2 h	Reference
			(MMD)						
Atogepant	NCT02848326	10 mg QD	−4.00 (10 mg QD)	57.6% (10 mg QD)	−3.71 (10 mg QD)	Not available	Not available	Not available	[20]
		30 mg QD	−3.76 (30 mg QD)	53.3% (30 mg QD)	−3.86 (30 mg QD)				
		30 mg BID	−4.23 (30 mg BID	52.0% (30 mg BID)	−3.77 (30 mg BID)				
		60 mg QD	−3.55 (60 mg QD)	58.2% (60 mg QD)	−3.53 (60 mg QD)				
		60 mg BID	−4.14 (60 mg BID)	62.1% (60 mg BID)	−3.64 (60 mg BID)				
Ubrogepant	NCT02828020	50 mg	Not available	Not available	Not available	19.2% (50 mg)	38.6% (50 mg)	Not available	[25]
		100 mg				21.2% (100 mg)	37.7% (100 mg)		
	NCT02867709	25 mg	Not available	Not available	Not available	20.7% (25 mg)	34.1% (25 mg)	Not available	[26]
		50 mg				21.8% (50 mg)	38.9% (50 mg)		
	NCT02873221	50 mg	Not available	Not available	Not available	24% (50 and 100 mg)	Not available	Not available	[27]
		100 mg							
	NCT01613248	1 mg	Not available	Not available	Not available	5.6% (1 mg)	Not available	37.40% (1 mg)	[29]
		10 mg				14.8% (10 mg)		52.8% (10 mg)	
		25 mg				21.4% (25 mg)		53.4% (25 mg)	
		50 mg				21.0% (50 mg)		57.1% (50 mg)	
		100 mg				25.5% (100 mg)		58.8% (100 mg)	
Zavegepant	NCT03872453	10 mg	Not available	Not available	Not available	22.5% (10 mg)	41.9% (10 mg)	Not available	[31]
		20 mg				23.1% (20 mg)	42.5% (20 mg)		
		5 mg				19.6% (5 mg)	39.0% (5 mg)		
Rimegepant	NCT01430442	10 mg	Not available	Not available	Not available	31.4% (75 mg)	Not available	Not available	[35]
		25 mg				32.9% (150 mg)			
		75 mg				29.7% (300 mg)			
		150 mg							
		300 mg							
		600 mg							
	NCT03461757	75 mg	Not available	Not available	Not available	21%	35%	Not available	[37]
Lasmiditan	NCT02439320	100 mg	Not available	Not available	Not available	28.2% (100 mg)	40.9% (100 mg)	59.4% (100 mg)	[42]
		200 mg				32.2% (200 mg)	40.7% (200 mg)	59.5% (200 mg)	
	NCT02605174	50 mg	Not available	Not available	Not available	28.6% (50 mg)	40.8% (50 mg)	59.0% (50 mg)	[43]
		100 mg				31.4% (100 mg)	44.2% (100 mg)	64.8% (100 mg)	
		200 mg				38.8% (200 mg)	48.7% (200 mg)	65.0% (200 mg)	
	NCT02565186	100 mg	Not available	Not available	Not available	26.7% (100 mg)	37.2% (100 mg)	Not available	[44]
		200 mg				32.2% (200 mg)	40.8% (200 mg)		

**Table 2:** Gepants and Ditans studies analyzed.

Drug	Identifier	Disease	Estimated Enrollment	Study Description	Intervention	Phase	Sponsors	Reference
Atogepant	NCT02848326	Migraine with or without aura	834	Safety and tolerability of Atogepant.	10 mg QD	2/3	Allergan	[20]
					30 mg QD			
					30 mg BID			
					60 mg QD			
					60 mg BID			
Ubrogepant	NCT02828020	Migraine with or without aura	1672	Efficacy, safety, and tolerability of 2 doses of ubrogepant.	50 mg	3	Allergan	[25]
					100 mg			
	NCT02867709	Migraine with or without aura	1686	Efficacy, safety, and tolerability of 2 doses of ubrogepant.	25 mg	3	Allergan	[26]
					50 mg			
	NCT02873221	Migraine with or without aura	1254	Evaluate the long-term safety and tolerability of ubrogepant over 1 year.	50 mg	3	Allergan	[27]
					100 mg			
	NCT01613248	Migraine	834	Assess the effectiveness, safety and tolerability of a range of doses of MK-1602 versus placebo.	1 mg	2	Allergan	[29]
					10 mg			
					25 mg			
					50 mg			
					100 mg			
Zavegepant	NCT03872453	Migraine	2154	Safety and efficacy of three different intranasal dose levels.	10 mg	2/3	Biohaven Pharmaceuticals	[31]
					20 mg			
					5 mg			
Rimegepant	NCT03235479	Migraine/Acute migraine	1485	Compare the efficacy of BHV-3000 vs. placebo in subjects with acute migraines.	75 mg	3	Biohaven Pharmaceuticals	[33]
	NCT03266588	Migraine with or without aura	3019	Evaluate safety and tolerability of BHV-3000.	75 mg	2/3	Biohaven Pharmaceuticals	[34]
	NCT01430442	Migraine	1026	Evaluate the efficacy of rimegepant compared with placebo in the acute treatment of migraine.	10 mg	2	Biohaven Pharmaceuticals	[35]
					25 mg			
					75 mg			
					150 mg			
					300 mg			
					600 mg			
	NCT03237845	Migraine Disorders	1499	Compare the efficacy of BHV-3000 vs. placebo in subjects with acute migraines.	75 mg	3	Biohaven Pharmaceuticals	[36]
		Photophobia						
		Phonophobia						
		Acute Migraine						
	NCT03461757	Migraine with or without aura	1811	Compare the efficacy of BHV-3000 vs. placebo in subjects with acute migraines.	75 mg	3	Biohaven Pharmaceuticals	[37]
Lasmiditan	NCT02439320	Acute Migraine	2231	A prospective study in participants with disabling migraine.	100 mg	3	Eli Lilly and Company	[42]
					200 mg		CoLucid Pharmaceuticals	
	NCT02605174	Migraine with or without Aura	3005	A prospective study in participants with disabling migraine.	50 mg	3	Eli Lilly and Company	[43]
					100 mg		CoLucid Pharmaceuticals	
					200 mg			
	NCT02565186	Migraine Disorders	2171	Safety Study of Lasmiditan in the acute treatment of migraine.	100 mg	3	Eli Lilly and Company	[44]
					200 mg		CoLucid Pharmaceuticals	
	NCT00883051	Migraine disorders	512	Efficacy and safety of a range of oral doses of	50 mg	2	Eli Lilly and Company	[46]
				COL-144 in treating migraine headache.	100 mg		CoLucid Pharmaceuticals	
					200 mg			
					400 mg			

## Data Availability

All the data sources are available within the article, have been properly cited and are publicly accessible.
